# Development of Web-Based Computer-Tailored Advice to Promote Physical Activity Among People Older Than 50 Years

**DOI:** 10.2196/jmir.1742

**Published:** 2012-03-02

**Authors:** Denise A Peels, Maartje M van Stralen, Catherine Bolman, Rianne HJ Golsteijn, Hein de Vries, Aart N Mudde, Lilian Lechner

**Affiliations:** ^1^Department of PsychologyOpen University of the NetherlandsHeerlenNetherlands; ^2^EMGO Institute for Health and Care ResearchDepartment of Public and Occupational HealthVU University Medical CentreAmsterdamNetherlands; ^3^Department of Health PromotionMaastricht UniversityMaastrichtNetherlands; ^4^Care and Public Health Research Institute (Caphri)Maastricht UniversityMaastrichtNetherlands

**Keywords:** Computer-tailored advice, physical activity, Web-based intervention, older adults, exercise, environment, RE-AIM model

## Abstract

**Background:**

The Active Plus project is a systematically developed theory- and evidence-based, computer-tailored intervention, which was found to be effective in changing physical activity behavior in people aged over 50 years. The process and effect outcomes of the first version of the Active Plus project were translated into an adapted intervention using the RE-AIM framework. The RE-AIM model is often used to evaluate the potential public health impact of an intervention and distinguishes five dimensions: reach, effectiveness, adoption, implementation, and maintenance.

**Objective:**

To gain insight into the systematic translation of the first print-delivered version of the Active Plus project into an adapted (Web-based) follow-up project. The focus of this study was on the reach and effectiveness dimensions, since these dimensions are most influenced by the results from the original Active Plus project.

**Methods:**

We optimized the potential reach and effect of the interventions by extending the delivery mode of the print-delivered intervention into an additional Web-based intervention. The interventions were adapted based on results of the process evaluation, analyses of effects within subgroups, and evaluation of the working mechanisms of the original intervention. We pretested the new intervention materials and the Web-based versions of the interventions. Subsequently, the new intervention conditions were implemented in a clustered randomized controlled trial.

**Results:**

Adaptations resulted in four improved tailoring interventions: (1) a basic print-delivered intervention, (2) a basic Web-based intervention, (3) a print-delivered intervention with an additional environmental component, and (4) a Web-based version with an additional environmental component. Pretest results with participants showed that all new intervention materials had modest usability and relatively high appreciation, and that filling in an online questionnaire and performing the online tasks was not problematic. We used the pretest results to improve the usability of the different interventions. Implementation of the new interventions in a clustered randomized controlled trial showed that the print-delivered interventions had a higher response rate than the Web-based interventions. Participants of both low and high socioeconomic status were reached by both print-delivered and Web-based interventions.

**Conclusions:**

Translation of the (process) evaluation of an effective intervention into an adapted intervention is challenging and rarely reported. We discuss several major lessons learned from our experience.

**Trial Registration:**

Nederlands Trial Register (NTR): 2297; http://www.trialregister.nl/trialreg/admin/rctview.asp?TC=2297 (Archived by WebCite at http://www.webcitation.org/65TkwoESp).

## Introduction

Regular physical activity reduces the risks of multiple health problems, which often become more prevalent when people age [1-3]. An international guideline for physical activity recommends that older adults should be physically active at moderate to vigorous intensity for at least 5 days a week, for a minimum of 30 minutes a day [4,5]. Regular physical activity is also particularly important for older adults to enable them to maintain their mobility and independence; to improve muscle strength, cognitive functioning, and mental and emotional well-being; and to prevent falls [2,4-8]. Because of the aging population in the Netherlands, stimulating physical activity among people over 50 years of age is of major relevance.

The Active Plus project is a computer-tailored, theory-driven, evidence-based intervention aimed at increasing physical activity in people aged over 50 years. The intervention, consisting of print-delivered tailored advice to improve the level of physical activity, has proven to be effective in changing physical activity behavior in the short and long terms, and was effective in reaching and affecting high-risk groups such as people of low socioeconomic status (SES) [9,10]. The purpose of this study was to gain insight into the translation of the first print-delivered version of the Active Plus project into an adapted (Web-based) follow-up project based on evaluation results, by using the RE-AIM framework.

The Active Plus project consisted of two phases: (1) systematic development and evaluation of the first version of the Active Plus project, which was completed in 2009 and has proven to be effective, and (2) translation of process and effect evaluation outcomes of the first Active Plus project into an adapted project using the RE-AIM framework to further increase the reach and effect of the project and its implementation in a clustered randomized controlled trial (RCT).

In the first phase of the Active Plus project (2005–2009), interventions were systematically developed using the intervention mapping protocol [11]. Development was based on a literature review [12], a Delphi study among experts in the field [13], focus group interviews, and a review of theoretical models, such as the I-Change Model [14], the Health Action Process Approach [15], self-regulation theory [16,17], and self-determination theory [18,19], to assess the most relevant and changeable personal and environmental determinants associated with changing physical activity behavior [20]. A complete description of the development and characteristics of the first version of the Active Plus project can be found elsewhere [20]. The first version of the Active Plus project consisted of two print-delivered, tailored physical activity interventions: (1) a basic computer-tailored intervention (to raise awareness of lack of physical activity, and to stimulate initiation and maintenance of physical activity), and (2) an intervention with additional environmental components that focused on giving tailored personalized advice on local possibilities and initiatives for being physically active for people over 50 years of age. The additional environmental component was intended to positively change people’s perceptions of the opportunities to be physically active in their own environment [21]. Both interventions included three tailored letters, based on the answers individuals gave in previous assessment, delivered by mail over 4 months [20]: (1) 2 weeks after the baseline assessment, (2) 2 months after the baseline assessment, and (3) 4 months after baseline assessment, based on the second assessment. The third tailored advice contained ipsative feedback about the changes in the respondents’ physical activity behavior and the psychosocial determinants in the previous 4 months. This means that improvements in (determinants of) physical activity were rewarded and possible relapses were addressed appropriately with additional suggestions to increase physical activity levels again.

Both original Active Plus interventions have proven effective in changing physical activity behavior [9,10]. Results showed that participants in the intervention groups increased their physical activity by 14%, from 4.2 days per week of at least 30 minutes of physical activity at baseline to 4.9 days per week at 6 months, while the control group did not change. At 12 months, the intervention groups were still physically active for 4.7 days per week. Further, both interventions were effective in reaching low-SES groups: 48% of all participants had a low level of education. Effects on physical activity were similar among different SES groups, but the low-SES group evaluated both interventions significantly more positively than did the high-SES group [9,22]. At 3 (ie, during the intervention), 6 (ie, 2 months postintervention), and 12 months (ie, 8 months postintervention), a process evaluation, an effect evaluation, and an evaluation of the working mechanisms of the intervention (ie, analyses of mediators) and less-responsive subgroups (ie, analyses of moderators) were conducted [10,22]. The results of these evaluations were the starting point for the second phase of this project.

In the second phase of the project, we translated the process and effect outcomes of the first version of the Active Plus project into an adapted intervention using the RE-AIM framework [23-26] and implemented the new interventions in a clustered RCT. The RE-AIM model is often used to evaluate the potential public health impact of an intervention [23]. The model distinguishes five different dimensions: (1) reach: the number and representativeness of individuals willing to participate, (2) effectiveness: the impact of the intervention on target outcomes, (3) adoption: the proportion and representativeness of settings that adopt the intervention, (4) implementation: the extent to which the interventions are delivered as intended, and (5) maintenance: the extent to which individual participants maintain behavior change in the long term and the extent to which the intervention is sustained over time within the organizations that deliver the intervention [23-26].

The aim of this study was to systematically translate the results of the (process) evaluation of the first version of the Active Plus project into an adapted follow-up (Web-based) project using the RE-AIM framework. Our focus was on the reach and effectiveness dimensions, since these dimensions are most influenced by the results from the original Active Plus project. Although relevant, the adoption, implementation, and maintenance dimensions depend on evaluation of the effectiveness of the new (Web-based) interventions, and were therefore beyond the scope of this paper. Additional evaluation results and response rates of the adapted interventions will be shown.

## Methods

Results of the process and effect evaluation of the original Active Plus project [10,21,22] were translated into a new (Web-based) project using the RE-AIM framework. We briefly outline the methods that we used to evaluate and adapt the Active Plus interventions according to the RE-AIM framework; we describe the actual adaptations in the Results section.

We improved the potential *reach* of the intervention by extending the delivery mode of the print-delivered intervention into an additional Web-based intervention with lower implementation costs. Furthermore, we used the process evaluation results of the original project [21] to detect how subgroups appreciated the intervention and thereby to subsequently adapt the interventions to optimize appreciation among certain groups and to increase the potential reach of the interventions.

To improve *effectiveness*, we used results of the process evaluation, analyses of effects within subgroups (ie, analyses of moderators [22]), and evaluation of the working mechanisms of the original intervention (ie, analyses of mediators [10,21]) to adapt and develop new intervention materials to eventually increase the potential effect of the intervention.

A quantitative pretest was performed using a questionnaire among 30 participants to study the appreciation and usability of all new intervention materials. Appreciation of the new intervention materials was assessed with 5 items per intervention element. Participants were asked whether they perceived the intervention materials as interesting, understandable, pleasant, advantageous, and motivating, using a 5-point Likert scale (ranging from 1, totally disagree, to 5, totally agree). We assessed usability using a 10-point Likert scale (ranging from 1, not useful, to 10, very useful). Additional in-depth open-ended questions were used to provide insight into the reasons why people gave certain ratings, and respondents were asked to write down their remarks regarding the new intervention strategies. The intervention strategies were subsequently adjusted according to the preferences of the pilot group.

A qualitative in-depth pretest (3 expert users and 7 nonexpert users) was performed to assess the usability of the new Web-based intervention as a whole. The expert users (all working in the field of health psychology) were asked to review the content of the intervention. All nonexpert participants were older than 50 years with little to average experience with Internet use, and were monitored while filling in the online questionnaire and reading the online advice. We used the results from this second pretest to further improve the Web-based intervention.

### Study Design

To study the effectiveness of the new interventions, and to monitor their adoption by the target population, we implemented the adapted interventions (a basic print-delivered intervention; a print-delivered intervention with environmental information; a basic Web-based intervention; and a Web-based intervention with environmental information) in a clustered RCT, including a control group that received no advice.

The main study parameters were changes in physical activity behavior (measured using the self-administered Dutch Short Questionnaire to Assess Health-Enhancing Physical Activity [27]), and determinants of changing and maintaining physical activity behavior.

### Participants and Procedure in the Clustered RCT

**Figure 1:**
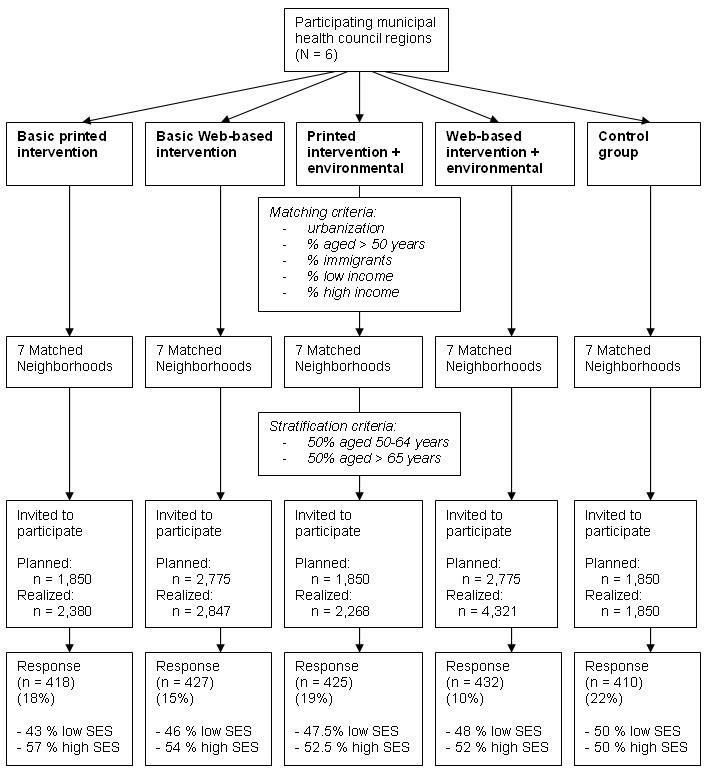
Flow diagram of the selection and response of participants. SES = socioeconomic status.

For the follow-up clustered RCT of the new interventions, we selected six municipal health council regions that had not participated in the first phase of the Active Plus project. To prevent participants from different intervention conditions contaminating each other (especially regarding whether they received environmental information), randomization was at the municipal health council region level, which means that each region was randomly assigned to one of the intervention conditions. This ensured that all participants were randomly assigned through their region to one of the conditions and were not able to choose the delivery mode (print delivered vs Web based) of the intervention. For each intervention condition, we selected seven (matched) neighborhoods to participate in the RCT. Neighborhoods were matched on their urbanization, percentage of people with a low SES, percentage of people with a high SES, percentage of immigrants, and percentage of people aged over 50 years. Each municipal health council provided a random sample of eligible participants living in the selected matched neighborhoods, after stratification for age. Figure 1 provides an overview of the selection of participants and of the number of participants for each intervention condition.

A power calculation (effect size = 0.4, power = 80%, intracluster correlation coefficient = .1) showed that at baseline about 420 participants were needed for each intervention condition (considering a dropout rate of 40% during the 1-year follow-up based on the original Active Plus project, and accounting for the multilevel design based on an estimate of the intracluster correlation coefficient). Because of an expected response rate of 23% (based on the previous Active Plus study [9]), our starting point was to distribute 1850 invitations in participating regions of the print-delivered intervention. Because the use of a Web-based intervention among persons aged over 50 years is less established, 2775 (50% extra) invitations were originally distributed in each Web-based intervention condition.

We recorded response rates for the different intervention conditions in SES groups.

This study was in accordance with the CONSORT checklist and is approved by the Medical Ethics Committee of Atrium–Orbis–Zuyd (METC code 10-N-36) and was registered with the Dutch Trial Register (NTR 2297).

**Figure 2:**
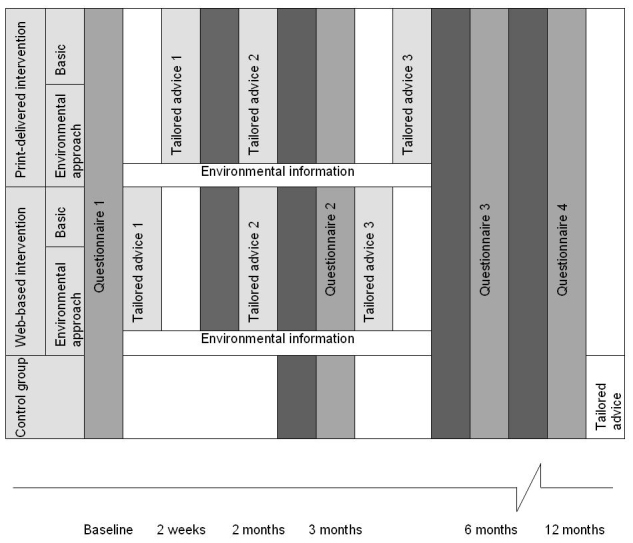
Timeline of the study.

The print-delivered intervention groups received their advice by mail, within 2 weeks after they returned the questionnaire. The Web-based intervention groups received an email, with a link that directly connected them with their tailored advice, immediately after filling in the questionnaire (see Figure 2 for the timeline of this clustered RCT).

## Results

This section describes the results of (1) the evaluation of the process and effect outcomes of the first version of the Active Plus project across the reach and effectiveness dimension of the RE-AIM framework, and (2) the adaptations in the intervention and delivery mode based on this process. Table 1 provides an overview of the results. Furthermore, this section shows the pretest results and response characteristics of the Active Plus follow-up project.

**Table 1:** Overview of applying the RE-AIM framework in evaluating and adapting the Active Plus project.

RE-AIM dimension	Definition	Adaptation for the Active Plus project
Reach	Number and representativeness of individuals willing to participate	Extending the delivery mode to a Web-based version
		Providing additional information to reach both low- and high-socioeconomic status groups
		Providing additional information to reach participants with physical disabilities
Effectiveness	Impact of the intervention on target outcomes	Translating the print-delivered interventions into Web-based interventions
		Adding information to increase intervention effects in the subgroups that were less affected by the original intervention (persons with a normal body mass index and persons aged over 65 years)
		Improving intervention strategies
		Stimulating self-efficacy and strategic planning
		Stimulating intrinsic motivation
		Reinforcing and extending environmental information
Adoption	Proportion and representativeness of settings that adopt the intervention	Translating the print-delivered interventions into Web-based interventions. Response rates to the print-delivered interventions are higher than response rates to the Web-based interventions^a^
Implementation	Extent to which the interventions are delivered as intended	^a^
Maintenance	Extent to which individual participants maintain behavior change long term and the extent to which the intervention is sustained over time within the organizations that deliver the intervention	Improvements made to the original interventions are expected to further increase the effects on physical activity behavior^a^

^a^ Further research is beyond the scope of the current study.

### Reach

Several efforts were made to increase the potential reach of the intervention. The delivery mode of the intervention was extended, and tailored advice was adapted to reach both low-and high-SES participants and to reach participants with physical disabilities.

#### Extending the Delivery Mode

The original Active Plus interventions were print delivered, distributed through written letters. In 2005 (the start of the original project) only a limited proportion of the older age groups had access to the Internet. However, in recent years Internet use among (older) adults has increased enormously. In 2010, 94% of all Dutch adults has access to the Internet at their home [28]. Although home Internet access is still lower in the age group 65+ years, with 68% access, the age group 55–65 years increased their home Internet access substantially to 91% in 2010. Furthermore, the differences in Internet access between SES groups have become much smaller (low SES 87%, high SES 98% in the general population), indicating that Web-based interventions are no longer a barrier to reaching low-SES groups or older adults [28].

Web-based tailoring offers several advantages over print-delivered tailoring, not only within the scope of reach but also in increasing the potential effects (which is the second dimension of the RE-AIM model and described in the next section). The main advantages of Web-based tailoring are that it provides options for more interactive applications (such as Google Maps, and meeting and discussion forums) and the opportunity to use more multimedia components (eg, physical activity options illustrated by videos, which should lead to better learning effects than static pictures [29]). Further, the Internet can deliver tailored feedback immediately, eliminating a time lag between assessment and feedback. The implementation costs of Web-based interventions are much lower, and they require less intensive manual labor than print-delivered interventions. Data from the print-delivered questionnaires need to be entered, and the letters need to be printed and sent by mail, which requires postage and portage. Translating the print-delivered interventions into Web-based interventions makes the interventions less expensive and less work intensive, and the project can be made more easily available to larger populations. Web-based interventions are therefore expected to have a larger reach. Other studies also showed that Web-based interventions have the potential to reach large audiences, with low costs per participant [30].

#### Reaching Low- and High-SES Groups

Both original interventions were effective in reaching low-SES groups: 48% of all participants had a low level of education [9,10]. As described above, it is expected that low-SES groups can be reached by a Web-based intervention. Furthermore, the process evaluation of the first project [21] showed that high-SES participants felt the need to receive more in-depth information about the effects of physical activity on health-related aspects. Therefore, we developed tailored messages with more in-depth information (such as the possible consequences of insufficient physical activity), especially for the more highly educated participants.

#### Reaching Participants With Physical Disabilities

Unpublished data from the first project showed that persons with physical disabilities had a lower appreciation of the original interventions. Because many people aged over 50 years have chronic physical disabilities (30% in the original Active Plus project), in the follow-up project we paid special attention to possible physical impairments of the participants. In the Netherlands, 38.1% of all people aged between 55 and 64 years have a chronic disease, increasing to 57.7% of all people aged above 75 years [31]. Therefore, being physically active with a chronic disease needs more attention, and the advice was tailored to certain physical disabilities. Participants with arthritis, cardiovascular diseases, depression, respiratory diseases, rheumatism, stroke, diabetes, osteoporoses, or severe back pain received additional tailored information about being physically active with their disability, since these disabilities have the highest prevalence among older persons in the Netherlands. This additional (existing) information was developed by the Dutch Institute for Sports and Movement and the Dutch Organization for Sports With Physical Impairments. 

### Effectiveness

To increase effectiveness, we improved the original Active Plus interventions by translating the print-delivered interventions into Web-based interventions, by targeting nonresponsive subgroups, and by improving the intervention strategies based on the results of the effect and process evaluation (see Table 1).

#### Web-Based Interventions

Because of several advantages, noted above, of Web-based interventions, we translated the print-delivered interventions into Web-based interventions. Research showed that Web-based interventions can be effective in changing health behaviors such as physical activity [32]. The Web-based interventions included more multimedia components. In the print-delivered Active Plus advice, a practical strategy to target modeling was to include role model statements in the letters, by using pictures of active similar others (same age and gender) combined with text describing the motivation and experiences of each person. For the Web-based version, we replaced these role model pictures and statements, as well as the traditional pictures to explain specific physical activity exercises, with short videos. Research has shown that video animations lead to better learning effects than static pictures [29].

We developed a website to provide the respondents with the opportunity to find information about physical activities in their own local surroundings, and to exchange information with other respondents. A meeting and discussion forum, an e-card facility, and Google Maps showing opportunities to be physically active in their own surroundings were developed specifically for participants in the environmental intervention conditions. We also added several hyperlinks to the website to provide participants with additional information about local physical activity possibilities. Participants in the Web-based intervention received an email with a copy (pdf format) of the personal advice they had received on the website directly after filling in the questionnaire. The advice sent by email had the same format as the print-delivered version.

#### Targeting Nonresponsive Subgroups

Moderation analyses showed that the interventions were not effective in persons with a healthy body mass index (<25 kg/m^2^) and adults 65 years and older [22]. Therefore, we added information on the advantages of physical activity for persons with a healthy body mass index and adults older than 65 years. The importance of physical activity when having a healthy weight was emphasized, and extra information was added about the benefits of physical activity especially for older adults, such as the importance of physical activity to maintain a healthy cognitive state.

#### Improving Intervention Strategies

We adapted some intervention strategies based on the process evaluation of the first project [21]. Regarding some of the original intervention strategies incorporated into the tailored letters (ie, role model statements, action planning post-its, and coping planning tables), the majority of the participants had read the materials and perceived the materials as interesting. However, when intervention components asked participants to actively participate (eg, filling in an action plan or coping plan), only a minority of them actually filled in the materials [9]. In the original interventions, action plans had to be written down on post-its. For this new intervention, the post-its were replaced by large and more appealing schemes, on which participants could plan and write down their weekly physical activities, with a more extensive and more motivating explanation. These schemes were integrated into several parts of the tailored advice, and participants were actively stimulated to work with these schemes.

#### Strategic Planning and Self-efficacy

Mediation analyses showed that strategic planning and self-efficacy were important in changing physical activity behavior [10]. Therefore, we provided more elaborated tailored advice on strategic planning in the improved interventions. The self-efficacy advice was formulated in a more empathetic way. Furthermore, more in-depth analyses showed that for some self-efficacy concepts (age, being physically active alone, and having less money), the transition from “being able to do it” to “being sure that they were able to do it” is of great importance. In the adapted interventions, the participants therefore also receive tailored text messages for these concepts when they answered “maybe” or “being able to do it,” whereas, in the original project, participants received self-efficacy messages only when they answered that they were “not (sure about) being able to do it.” In addition, participants received a logbook with instructions to encourage them to monitor their own physical activity behavior and thereby to increase their awareness of such behavior and to stimulate maintenance of being physically active.

#### Intrinsic Motivation

Mediation analyses of the original Active Plus project showed that the intervention had only a minimal effect on the participants’ intrinsic motivation to be physically active [10]. By integrating some concepts of the self-determination theory into the current intervention, we attempted to increase the participants’ intrinsic motivation. The self-determination theory emphasizes the importance of intrinsic motivation in engaging in and maintaining health-related behaviors. Motivation is autonomous or self-determined when a person’s perceived locus of causality is internal. Intrinsic motivation implies that the behavior is engaged in for enjoyment and satisfaction [33-35]. Several studies have shown the importance of intrinsic motivation as a predictor in exercise participation [36-40]. People will develop and maintain more self-determined motivation when their environment supports autonomy. This implies that people’s perspective should be acknowledged, their initiatives should be supported, a choice and relevant information should be provided, and pressure and control should be minimized [33-35]. To create an autonomy-supportive environment, we adapted text messages to comply with the self-determination theory [34]. A clear rationale to adopt the behavior was provided by presenting a more elaborated knowledge component about the contingencies between behavior and health outcomes to support informed choices. Also, participants’ feelings and perspectives were acknowledged more by showing more empathy. Participants were stimulated to think and write down their personal most important reasons to be sufficiently physically active. Furthermore, the participants’ own initiatives were more supported. For example, on every improvement, positive feedback was given even when the physical activity behavior still did not comply with the international physical activity guidelines. Participants were also offered a choice of options giving them control over their own physical activity behavior (eg, physical activity in their daily activities, during their leisure time, or by exercising). Finally, we checked all text messages for the use of neutral language: “should” and “must” were replaced by “may” and “could.”

#### Reinforcing and Extending Environmental Information

Previous studies have emphasized the importance of focusing on enhancing participants’ perceptions of their environment [41-47]. Unfortunately, we found no overall differences in physical activity effects between the environment-based Active Plus intervention and the basic tailored intervention [10,21]. However, perceptions of the environment changed positively: participants perceived significantly more opportunities to engage in physical activity in their immediate environment. Furthermore, at 6 months the intervention additionally focusing on environmental aspects increased the amount of certain types of physical activities such as walking and cycling more than did the basic intervention [10,21]. Mediation analyses showed that the positive effect of the environmentally tailored intervention on physical activity was mediated by changes in environmental perceptions [10,21]. This emphasizes the importance of including aspects such as enhancing participants’ perceptions of their environment in tailored interventions. Adding environmental information to the intervention is supposed to further increase the intervention effects. Therefore, an extended version of the environment-based intervention was developed for this follow-up project.

In the original Active Plus project, participants in the environmental intervention conditions received information on walking and cycling routes in their own neighborhoods, written examples of exercises to do at home, contact information for neighborhood sport clubs, a roadmap of their immediate neighborhood on which walking and cycling possibilities were highlighted, and access to a forum and e-buddy system on a website. In the adapted follow-up intervention, we emphasized the environmental approach, making it more personalized and extended with additional options. Information about local opportunities to be physically active was added on three levels: (1) in their own district (sport clubs or sport accommodations matching with their own preferences, existing walking and cycling routes, and options to develop their own walking and cycling routes), (2) in their immediate neighborhood (walking routes to stores within 1 km of their own house, using Google Maps), and (3) in their own home (options for being more physically active in their daily life activities and seven different home exercises).

Furthermore, we paid more attention to the participants’ social environment. Although the original environmental Active Plus intervention contained an option to visit a website to contact other adults to be physically active together, little emphasis was placed on this opportunity, resulting in very low participation. In the new intervention, we put greater emphasis on opportunities to get together with others in the neighborhood to be physically active. We developed an online forum with a variety of topics, actively promoted the use of a well-known website to find a sport mate (www.beweegmaatje.nl), and developed several postcards and e-cards that the participant could use to invite someone to be physically active together. Participants also received tailored feedback about the social modeling and social support they receive from their immediate social environment.

##### Pretest

All of the new intervention materials were quantitatively and qualitatively evaluated for appreciation and usability. Results of the quantitative pretest showed (see Table 2) that the new strategies had modest usability (scores between 5.3 and 6.4 on a scale of 1, not useful, to 10, very useful) and relatively high appreciation (scores between 3.7 and 4.1 on a scale of 1, low appreciation, to 5, high appreciation). Based on these results and the remarks of the participants on the materials, we subsequently adjusted the intervention strategies according to the preferences of the pilot group.

The qualitative pretest assessing the usability of the Web-based intervention (see Table 3) showed that filling in an online questionnaire and performing the online tasks (such as sending e-cards, filling in pdf forms, or watching videos) was not problematic for the nonexpert users. Although filling in the questionnaire and reading the advice took a considerable amount of time, the participants did not mention this as a constraint.

**Table 2:** Summary of quantitative pretest results.

Determinant	Theoretical method	Intervention strategy	Intervention materials	Appreciation score (1–5)	Usability score (1–10)
Action planning	Active learning	Invite to formulate action plan	Weekly schema to write down plans to be physically active (when, what, where, with whom)	3.89	5.93
Coping planning	Active learning	Invite to formulate coping plans	Coping planning schema	3.72	5.78
Perceived social environment or having a sports partner	Linking members to networks of people	Provide the opportunity to contact others	Post card to invite someone to be physically active together	3.96	5.32
Perceived physical environment	Facilitating	Provide exercises to do at home	Physical activity exercises	4.06	6.64
Awareness	Self-monitoring	Encourage monitoring of own behavior	Logbook	3.73	5.79

**Table 3:** Summary of qualitative pretest results of the Web-based intervention.

General findings	Adaptations
The website address is often typed in into the Google search window instead of the website address window.	The website was made findable in the Google search results, and an information form was developed on how to enter the website.
Average duration of filling in the online questionnaire was 50 minutes. The participants did not mention this as a constraint.	No adaptations were made to the length of the questionnaire.
People perceived difficulties in how to fill in certain questions.	An information form was developed on how to fill in this questionnaire. This information was also added to the help section of the website.
People appreciated the large letter type.	The letter type size was not changed.
All people were able to use the scrolling methods, but scrolling both up and down, and to the left and to the right is too difficult.	Layout of the webpage was adapted to prevent too much scrolling.
By clicking on other website links, participants lost track of their own tailored advice.	Website links mentioned in the advice opened in a new window, to prevent participants from loosing track of their personal advice.
Some technical errors and errors in tailoring algorithms were found.	All errors were adjusted.

### Adoption, Implementation, and Maintenance

To study effectiveness and to monitor adoption of the interventions by the target population, the interventions were implemented in a clustered RCT. Since the response percentages were lower than expected, in a second recruitment round we sent additional invitations to reach similar absolute numbers of respondents in all intervention groups. Figure 1 shows that the response rate to the print-delivered intervention was higher than the response rate to the Web-based intervention among people aged over 50 years. As expected, both low- and high-SES participants were reached by both print-delivered and Web-based interventions.

Changes made in the intervention strategies to increase the intervention effects (eg, stimulating intrinsic motivation) were also expected to further increase the maintenance of physical activity behavior among the target population.

## Discussion

This paper describes the systematic translation of the results of the (process) evaluation of the original version of the Active Plus project into an adapted (Web-based) intervention using the RE-AIM framework. Our focus was on the reach and effectiveness dimensions of the RE-AIM framework, since these dimensions are most influenced by the results from the original Active Plus project.

We optimized the potential reach and effect of the Active Plus interventions by translating the print-delivered interventions into Web-based interventions. We also improved the potential reach of the intervention by adapting the tailored advice to reach both low- and high-SES participants and to reach participants with physical disabilities. To further increase the effectiveness of the original Active Plus interventions, we adapted them to appeal to subgroups that did not respond to the effect in the original intervention (ie, persons with a healthy body mass index and adults older the 65 years). Furthermore, several intervention strategies were improved. Because results from the original project showed that strategic planning, self-efficacy, intrinsic motivation, and the environmental component were important in changing physical activity behavior, these concepts were more strongly targeted in the new interventions. We made the adaptations based on the results of the first Active Plus project, such as the process evaluation [21], analyses of effects within subgroups (ie, analyses of moderators [22]), and evaluation of the working mechanisms of the original interventions (ie, analyses of mediators [10,21]).

Pretest results showed that all of the new intervention materials had modest usability and relatively high appreciation, and that filling in an online questionnaire and performing the online tasks was not problematic for adults aged over 50 years. We subsequently adjusted the intervention strategies according to the preferences of the pilot group and then implemented the interventions in a clustered RCT. Our first results showed a higher response rate for the print-delivered interventions than for the Web-based interventions. However, different SES groups were equally reached.

We have identified several major lessons from our experience of translating the original intervention into a Web-based intervention targeted at older adults. First, it is essential to use a theoretical framework such as the RE-AIM model when evaluating and adapting an original intervention, since it ensures that all important points that can determine the impact of an intervention are systematically addressed. Second, it is of major importance to use process evaluation data, and mediation and moderation results to redesign and strengthen an effective intervention. Finally, it is imperative to thoroughly pretest the new interventions. The combination of quantitative and qualitative pretests used in this study was very useful to gain a broad insight into user experiences and preferences, and thereby to improve the usability of the intervention.

Translating the (process) evaluation of an effective intervention into an adapted intervention is very challenging and rarely reported. Evaluation studies seldom lead to adaptations in the evaluated interventions, at least not ones that are scientifically monitored and reported, which results in a gap between research insights and practice. Evidence on how to effectively facilitate this adaptation process is also limited [48]. Providing more insight into intervention evaluations and adaptations could help future research in systematically (re)evaluating and (re)designing interventions, without reinventing the wheel.

Our adaptations led to four versions of the intervention: basic or with additional environmental information, and print delivered or Web based. In future evaluations, it will be important to consider the differences in cost and effects of the different interventions as well. The potential reach of the Web-based interventions is expected to be higher than the print-delivered intervention because of the lower implementation cost. Furthermore, the effect of the Web-based intervention on behavioral change might be better due to the advantages of the Internet (eg, the possibility of immediate feedback and multimedia use). Response rates showed that both low- and high-SES participants can be reached by both print-delivered and Web-based interventions. The continuing study of the Active Plus project aims to provide more insights into the cost effectiveness of the adapted interventions.

The RE-AIM framework was perceived as a very useful guideline for evaluating the original intervention and translating it into a new intervention. We emphasized the reach and effectiveness dimensions in our study. However, gaining insight into the most relevant conditions for the adoption and implementation of an intervention is also of major importance and will be the focus of future research. The adoption, implementation, and maintenance dimensions would depend on the cost-effectiveness evaluation of the new (Web-based) interventions, and will therefore be studied in a later stage of this project. Results of the effect evaluation of the new interventions and experiences of the target population and intermediaries with the intervention may serve as input for future studies. Careful administration of all costs and time needed for the development, recruitment, and implementation of the interventions is important to enable different intermediaries to judge whether they would have the capacity to implement these interventions in a nonexperimental setting.

Future studies should also provide insight into the feasibility and relevant conditions for accepting and implementing the interventions (by the intermediaries) or for participating in the interventions (target group). Since Web-based interventions among persons aged over 50 years can be regarded as an innovation, a study to inform the adaption, implementation, and maintenance of the intervention could be guided by Rogers’ theory of innovations [49], for example, by using focus group interviews or in-depth interviews.

This paper provides insight into the processes leading to future effects of the intervention and could help future researchers in systematically developing or reevaluating their intervention.

## Abbreviations

RCT: randomized controlled trial
SES: socioeconomic status
